# Cardiac progenitor cells for heart repair

**DOI:** 10.1038/cddiscovery.2016.52

**Published:** 2016-07-18

**Authors:** TYL Le, JJH Chong

**Affiliations:** 1 Centre of Heart Research, Westmead Institute for Medical Research, The University of Sydney, Hawkesbury Road, Westmead, Sydney, New South Wale 2145, Australia; 2 Department of Cardiology, Westmead Hospital, Hawkesbury Road, Westmead, Sydney, New South Wale 2145, Australia; 3 Sydney Medical School, The University of Sydney, Sydney, New South Wale 2006, Australia

## Abstract

Stem cell therapy is being investigated as an innovative and promising strategy to restore cardiac function in patients with heart failure. Several stem cell populations, including adult (multipotent) stem cells from developed organs and tissues, have been tested for cardiac repair with encouraging clinical and pre-clinical results. The heart has been traditionally considered a post-mitotic organ, however, this view has recently changed with the identification of stem/progenitor cells residing within the adult heart. Given their cardiac developmental origins, these endogenous cardiac progenitor cells (CPCs) may represent better candidates for cardiac cell therapy compared with stem cells from other organs such as the bone marrow and adipose tissue. This brief review will outline current research into CPC populations and their cardiac repair/regenerative potential.

## Facts

- The adult heart is capable of limited cardiomyocyte turn over, enhanced directly or indirectly by endogenous cardiac progenitor cells.
- The specific biological roles of cardiac progenitor cell populations in the injured heart still remains elusive.
- Favourable effects of cardiac progenitor cells after transplantation into injured myocardium is likely due to multiple mechanisms including neovascularisation and favourable remodelling of cardiac scar.

## Open Questions

- Which cardiac progenitor cell populations (c-Kit+, Sca-1+, Islet-1+, cardiosphere-derived cells, side population cells, epicardium derived cells and c-CFU-Fs) play a predominant role in repairing the damaged heart?
- What is the overlap between the several cardiac progenitor cell populations listed above?
- Do cardiac progenitor cells contribute directly to new adult cardiomyocytes lost after myocardial injury?

Cardiovascular disease continues to be a major cause of morbidity and mortality worldwide. Despite considerable progress in revascularisation techniques and pharmacotherapy, many patients progress to heart failure after acute myocardial infarction (MI). Current therapies are unable to replace dead cardiomyocytes (CMs) and largely irreversible cardiac dysfunction ensues. The discovery of multiple classes of stem cells has generated hope for their use as therapeutic agents in heart failure. This may involve the transplantation of pluripotent stem cells or multipotent adult progenitor cells into the area of the infarcted myocardium, to promote regeneration of new functioning myocytes and vascular cells, thus improving heart function.

The adult mammalian heart was previously considered a post-mitotic organ without the capacity for self-renewal. However, recent evidence suggests that the adult heart is capable of CM turn over, possible from endogenous cardiac stem cells/cardiac progenitor cells (CPCs).^1,2^ In a genetic fate-mapping study, it was shown that CPCs contribute to the replenishment of adult mammalian CMs lost after injury, throughout the adult life.^3,4^ Numerous other studies have also identified and isolated endogenous CPC populations in the hearts from multiple species including rodents, dogs, pigs and humans.^5–10^ These studies suggest that CPCs are capable of differentiating into multiple cell types, such as CMs, vascular smooth muscle cells and endothelial cells (ECs). Intra-myocardial transplantation of CPCs after induced MI, resulted in reduction of scar size and improvement in left ventricular (LV) function, have been demonstrated in pre-clinical models. These findings promised a paradigm shift in cardiac biology and new opportunities for future treatment. However, 15 years after these initial reports, a consensus on the biological role of these populations still remains elusive. In this review, we provide a brief overview of the CPCs currently being considered for cardiac repair and the potential mechanisms of action of CPCs in the damaged heart.

## Cardiac Progenitor Cells

**Figure 1:**
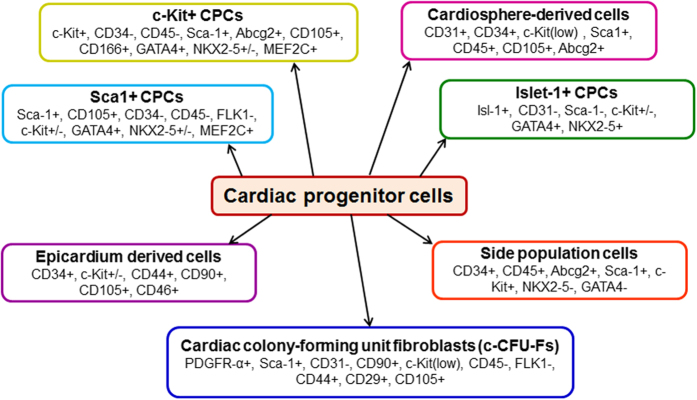
Summary of endogenous CPC populations.

**Figure 2:**
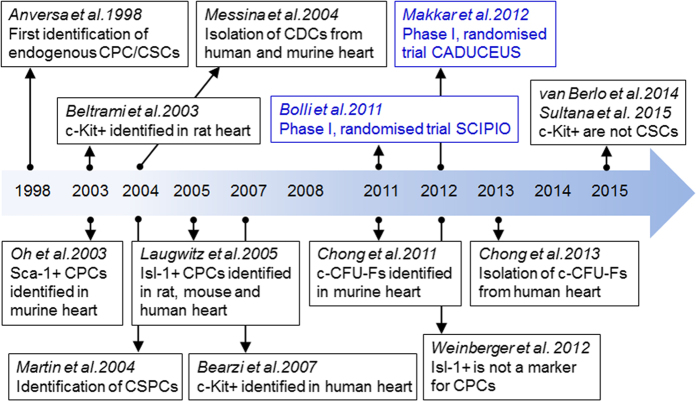
Timeline for major events in studies of the cardiac stem/progenitor cells. CADUCEUS, cardiosphere-derived autologous stem cells to reverse ventricular dysfunction; CSPC, cardiac side population cell; SCIPIO, stem cell infusion in patients with ischaemic cardiomyopathy. Randomised trials of CPCs as therapy are indicated in blue.

CPCs are a heterogeneous group of cells distributed throughout the heart, (including the atria, ventricles, and epicardium or pericardium). Under normal physiological conditions, CPCs are thought to be quiescent and do not contribute significantly to CM renewal.^3,4^ After injury, however, CPCs can be activated and may differentiate into new myocytes or vascular cells.^3,4^ Unlike other adult cell types such as bone marrow cells (BMCs), for which surface markers have been extensively characterised, resident CPCs show mixed and overlapping expression of stem cell markers (Figure 1). Several CPC populations have been reported in the developing and adult heart including: c-Kit^+^ CPCs; cardiospheres/cardiosphere-derived cells (CDCs); epicardium derived cells; cardiac side population cells (identified by their ability to exclude Hoechst dye from nuclei^8^); stem cell antigen-1 (Sca-1^+^) CPCs; Islet-1 (Isl-1^+^) expressing CPCs and platelet derived growth factor receptor-alpha (PDGFRα^+^) expressing CPCs (interested readers are directed to other reviews for further detail^11,12^). A timeline for major events in studies of cardiac stem/progenitor cells is shown in Figure 2. Although considerable overlap between these populations is likely, there is insufficient data to specifically address this possibility.

CPCs were first identified in 2003 through the expression of the tyrosine kinase receptor, c-Kit and the absence of common hematopoietic lineage markers (such as CD45, CD34, CD3, CD14, CD16, CD19, CD20 and CD56) in the adult mammalian heart.^13^ c-Kit^+^ cardiac cells possess prolonged self-renewing, clonogenic and multipotent characteristics. Encouraging results whereby c-Kit^+^ CPCs improved LV dysfunction and remodelling in various pre-clinical models of post-MI cardiomyopathy have paved the way for Cardiac Stem cell Infusion in Patients with Ischemic Cardiomyopathy (SCIPIO), the first clinical trial of CPCs. In SCIPIO, c-Kit^+^ cells were isolated from the right atrial appendage of patients undergoing open heart surgery for coronary artery bypass grafting. Harvested CPCs underwent expansion *in vitro* and were then infused back into the donor heart via the coronary arterial circulation. Although the SCIPIO results appeared to support pre-clinical work by the same group (improvement in LV systolic function and reduced infarct size,^14,15^ it is important to note that editors of the prestigious *Lancet* journal have taken the unusual step of expressing concern over the integrity of data published in the SCIPIO trial.^16^

Another well-characterised CPC population is the CDC population. CDCs are a mixture of stromal, mesenchymal and progenitor cells that are derived from cultures of atrial or ventricular biopsy specimens. When cloned in suspension, they develop multicellular clusters known as cardiospheres.^17^ From these cardiospheres, millions of proliferative cells that express markers of mesenchymal, and progenitor cell-related antigens can be harvested.^17^ *In vitro* CDCs are clonogenic and have multilineage potential. The safety and efficacy of CDC transplantation has also been demonstrated in pre-clinical studies.^10^ In a murine model of MI, the functional outcome of CDC transplantation was superior (in terms of ischaemic tissue preservation, positive remodelling and functional benefits) to the transplantation of bone marrow mesenchymal stromal cells and adipose-derived regenerative cells.^18^ In the cardiosphere derived autologous stem cells to reverse ventricular dysfunction (CADUCEUS) study, patients with acute MI were randomised to receive standard medical therapy or autologous CDCs.^19^ This phase I randomised clinical trial showed no difference in LV ejection fraction after 6 months.^19^ However, infarct size was significantly reduced in the cell treated group. Patients treated with CDCs showed a reduction in scar mass, increased viable heart mass and improved regional contractility.^19^

Several other CPC populations have also been identified and characterised using different membrane markers (Sca-1, Abcg-2, Flk-1, CD34, CD90 and CD105) and transcription factors (Isl-1, Nkx2.5, MEF2C and GATA4) in the embryonic and adult heart (Figure 1). Again, these cells are clonogenic, self-renewing and multipotent both *in vitro* and *in vivo*. They express several markers characteristic of stem cells (Oct3/4, Bmi-1 and Nanog) and have significant regenerative potential *in vivo*. However, recent evidence has suggested that Isl-1 is not a marker of endogenous CPCs.^20^ Furthermore, using genetic fate-mapping approaches, Isl-1 has been shown to mark not only progenitors from the second heart field, but also from the cardiac neural crest.^21^ Recent reports suggest that resident cardiac c-Kit^+^ cells in the mouse are not a source of myocytes but are predominantly a source of ECs after cardiac injury.^22,23^ Therefore, as previously mentioned, the exact biological role of these various progenitor populations in the injured heart remains unknown.

**Figure 3:**
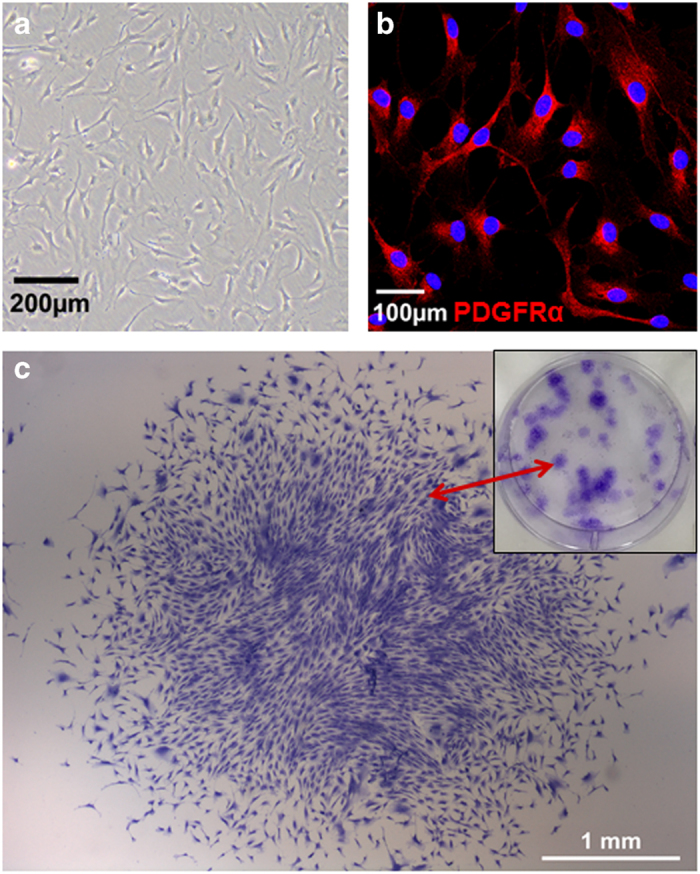
Cardiac c-CFU-F population. (**a**) Representative image of the c-CFU-Fs isolated from human heart tissue, (**b**) c-CFU-Fs expressed the tyrosine kinase PDGFRα^+^ and (**c**) representative image of crystal-violet stained c-CFU-F colonies.

We have recently demonstrated that expression of the tyrosine kinase PDGFRα^+^ identifies a resident cardiac progenitor population (named colony-forming unit fibroblasts; c-CFU-Fs) in the murine^6^ and human heart^24^ (Figure 3). These cells possess prolonged self-renewal and multipotent potential *in vitro*. CRE lineage-tracing studies suggest a proepicardial/epicardial origin for c-CFU-Fs.^6^ Both cardiac fibroblasts and c-CFU-Fs originate from the proepicardium, and undergo epithelial-mesenchymal transition before populating the subepicardium and myocardial interstitium, where they adopt a perivascular location.^6^ Under specific conditions c-CFU-Fs are able to give rise to vascular cell types, differentiated fibroblasts and to a more limited degree, CM-like cells. We are currently continuing further studies in rodent models of cardiac injury that will lead to a better understanding of the pathophysiological role of these progenitors.

## Potential Mechanisms of Cardiac Progenitor Cells Action in the Damaged Heart

**Figure 4:**
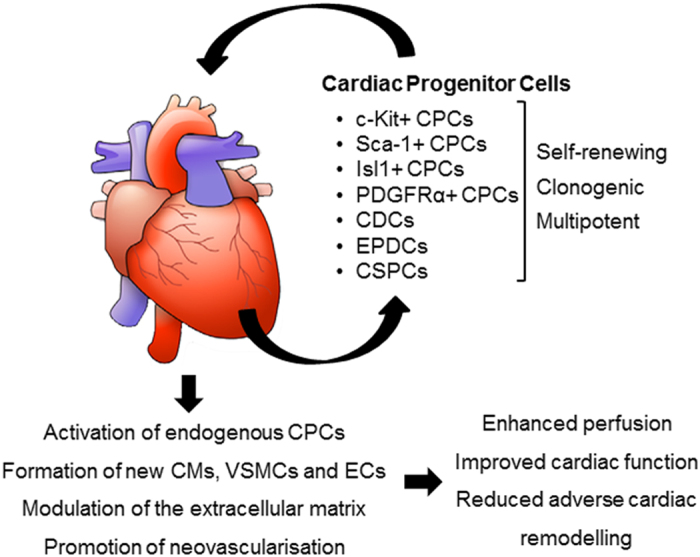
Potential mechanisms of action of transplanted CPCs. CSPC, cardiac side population cell; EPDC, epicardium derived cell; VSMC, vascular smooth muscle cell.

Implantation of CPCs in the injured heart may lead to myocardial repair via direct and indirect mechanisms (Figure 4). These include directly transdifferentiation into CMs and vascular cells, secretion of paracrine factors inducing hyperplasia proliferation of existing CMs, inducing resident endogenous CPCs differentiation and cell fusion between transplanted cells and existing CMs. After many years of pre-clinical and clinical studies, the predominant view is that the positive effects of adult stem cell therapy (such as BMC and adipose-derived cells) is mediated through paracrine release of anti-apoptotic, immunomodulatory, proangiogenic host- and cell-derived factors.^25^ Therefore, the moderate positive effects of adult stem cell delivery to impaired myocardium is more likely due to enhanced neovascularisation or favourable changes in the cardiac scar (which is itself contractile and not inert), rather than the formation of new CMs. Direct transdifferentiation into CMs (as robustly seen in current pluripotent stem cell differentiation protocols) is considered unlikely.

Many of the pre-clinical studies and the SCIPIO clinical trial (discussed above) have used c-Kit^+^ cells as the primary source of cardiac regeneration after injury and a more recent study has used powerful genetic fate-mapping experiments to show CM formation from c-Kit^+^ progenitors in a rodent catecholamine-induced injury model.^26^ However, van Berlo *et al*.^22^ have also used genetic fate-mapping experiments to arrive at a contrary conclusion, that minimal CMs are generated from c-Kit^+^ cells *in vivo*. In contrast, abundant cardiac ECs appeared to be derived from c-Kit^+^ CPCs.^22,23^ Taken together, these new findings suggest that improvements in cardiac function after injury may be due to c-Kit^+^ CPC vascularisation of the injured heart and subsequent favourable effects on hibernating myocardium rather than the generation of new CMs.

## Future Directions

Despite publication of many studies in multiple different species, the physiological and pathophysiological functions of the various CPC populations in the heart remain unclear. Utilisation of CPCs (either exogenously delivered or harnessing CPCs residing within the injured heart) has repeatedly been shown to induce favourable reparative or regenerative effects. Nevertheless the molecular mechanisms underpinning such changes are also not completely understood. Thus, the application of CPCs as a clinical treatment for cardiovascular disease will remain difficult until these limitations are appropriately addressed. In addition, attention should be focused on gaining a better understanding of the cardiac fibroblast population, which is known to aid repair and regeneration of CMs in addition to producing cardiac scar. Currently, what defines the cardiac fibroblast pool is poorly understood.^27^

## Abbreviations

CPC: cardiac progenitor cell
CDC: cardiosphere-derived cell
CSPC: cardiac side population cell
c-CFU-F: cardiac colony-forming unit fibroblast
EPDC: epicardium derived cell
BMC: bone marrow cell
PDGFRα: Platelet Derived Growth Factor Receptor-alpha
Sca-1: stem cell antigen-1
MI: myocardial infarction
LV: left ventricular
CM: cardiomyocyte
EC: endothelial cell
VSMC: vascular smooth muscle cell
CADUCEUS: CArdiosphere Derived aUtologous Stem CElls to Reverse ventricUlar dysfunction
SCIPIO: Stem Cell Infusion in Patients with Ischemic cardiomyopathy.
